# Activation of the SARS-CoV-2 NSP14 3′–5′ exoribonuclease by NSP10 and response to antiviral inhibitors

**DOI:** 10.1016/j.jbc.2021.101518

**Published:** 2021-12-20

**Authors:** Amanda A. Riccio, Eric D. Sullivan, William C. Copeland

**Affiliations:** Genome Integrity and Structural Biology Laboratory, National Institute of Environmental Health Sciences, NIH, Research Triangle Park, North Carolina, USA

**Keywords:** enzyme kinetics, protein purification, protein stability, protein–protein interaction, viral protein, protein complex, protein drug interaction, SARS-CoV-2, coronavirus, COVID-19, NSP14, NSP10, viral proofreading, exoribonuclease, COVID-19, coronavirus disease 2019, CV, column volume, DDEE, residues forming the active site aspartic acid (D) and glutamic acid (E), DMSO, dimethyl sulfoxide, DSF, differential scanning fluorimetry, FL, full-length, MP, monophosphate, MTase, methyltransferase, Ni, nickel, NP-40, Nonidet-P40, NSP14, nonstructural protein 14, NTA, nitrilotriacetic acid, RMP, remdesivir monophosphate, SAH, S-adenosylhomocysteine, SARS-CoV-2, severe acute respiratory syndrome coronavirus 2, SEC, size-exclusion chromatography, TCEP, Tris(2-carboxyethyl)phosphine

## Abstract

Understanding the core replication complex of severe acute respiratory syndrome coronavirus 2 (SARS-CoV-2) is essential to the development of novel coronavirus-specific antiviral therapeutics. Among the proteins required for faithful replication of the SARS-CoV-2 genome are nonstructural protein 14 (NSP14), a bifunctional enzyme with an N-terminal 3′-to-5′ exoribonuclease (ExoN) and a C-terminal N7-methyltransferase, and its accessory protein, NSP10. The difficulty in producing pure and high quantities of the NSP10/14 complex has hampered the biochemical and structural study of these important proteins. We developed a straightforward protocol for the expression and purification of both NSP10 and NSP14 from *Escherichia coli* and for the *in vitro* assembly and purification of a stoichiometric NSP10/14 complex with high yields. Using these methods, we observe that NSP10 provides a 260-fold increase in *k*_cat_/*K*_*m*_ in the exoribonucleolytic activity of NSP14 and enhances protein stability. We also probed the effect of two small molecules on NSP10/14 activity, remdesivir monophosphate and the methyltransferase inhibitor S-adenosylhomocysteine. Our analysis highlights two important factors for drug development: first, unlike other exonucleases, the monophosphate nucleoside analog intermediate of remdesivir does not inhibit NSP14 activity; and second, S-adenosylhomocysteine modestly activates NSP14 exonuclease activity. In total, our analysis provides insights for future structure–function studies of SARS-CoV-2 replication fidelity for the treatment of coronavirus disease 2019.

Severe acute respiratory syndrome coronavirus 2 (SARS-CoV-2) is the causative agent of the current coronavirus disease 2019 (COVID-19) worldwide pandemic. SARS-CoV-2 is an RNA virus belonging to the Coronaviridae family. The Coronaviridae virus family was also responsible for the 2000 to 2004 SARS outbreak and the Middle East respiratory syndrome, first detected in 2012 (1, 2). At the time of this publication, SARS-CoV-2 infection has resulted in 4.3 million deaths worldwide and sweeping economic shutdowns and continues to be transmitted at high rates (3). Current therapeutics for the treatment of COVID-19 are limited. To date, the American Food and Drug Administration has approved only a single drug, Gilead Sciences Veklury (remdesivir), for the treatment of COVID-19 (4). Remdesivir is a nucleoside analog, based on a scaffold used to produce antiviral treatments for hepatitis B and C, as well as HIV, and was originally developed for the treatment of Ebola virus infections (5, 6, 7). Since remdesivir belongs to a broad-spectrum class of antiviral drugs that inhibit viral RNA synthesis, more targeted therapeutics for the treatment of COVID-19 are needed.

The SARS-CoV-2 RNA genome encodes 29 proteins, of which 16 are nonstructural proteins (NSPs) required to carry out the viral replication cycle (8). In the treatment of coronaviruses, incorporation of nucleoside analogs, such as remdesivir, causes a delayed chain termination that results in aborted RNA synthesis. This activity is balanced with the excision of the analogs through 3′–5′ proofreading exoribonucleolytic activity, promoting potential means of resistance (9, 10). The key components of SARS-CoV-2 replication machinery are the RNA-dependent RNA polymerase complex, which includes the RNA polymerase, NSP12, and the processivity factors NSP7 and NSP8, as well as the proofreading nuclease, NSP14 and its coactivator, NSP10. Recent structural analysis highlights a network of the interdomain contacts between NSP14, NSP10, and NSP12 in the context of a capping and proofreading complex (11, 12), yet the molecular mechanisms that define these interactions are still under investigation. NSP14 is a bifunctional enzyme characterized by an N-terminal exoribonuclease domain named for the catalytic residues in the superfamily (DEDD [residues forming the active site aspartic acid (D) and glutamic acid (E)]) and a C-terminal methyltransferase (MTase) domain, which catalyzes N7-guanosine methylation (Fig. 1*A*) (13, 14). NSP10 is a 139 amino acid protein that is an activator of both NSP14 and NSP16, a 2′-O-MTase, which forms a crucial step in capping nascent mRNA (15). In addition to sharing a coactivator, NSP14 and NSP16 are both S-adenosyl-l-methionine-dependent MTases and generate S-adenosylhomocysteine (SAH) as a reaction byproduct.

**Figure 1:**
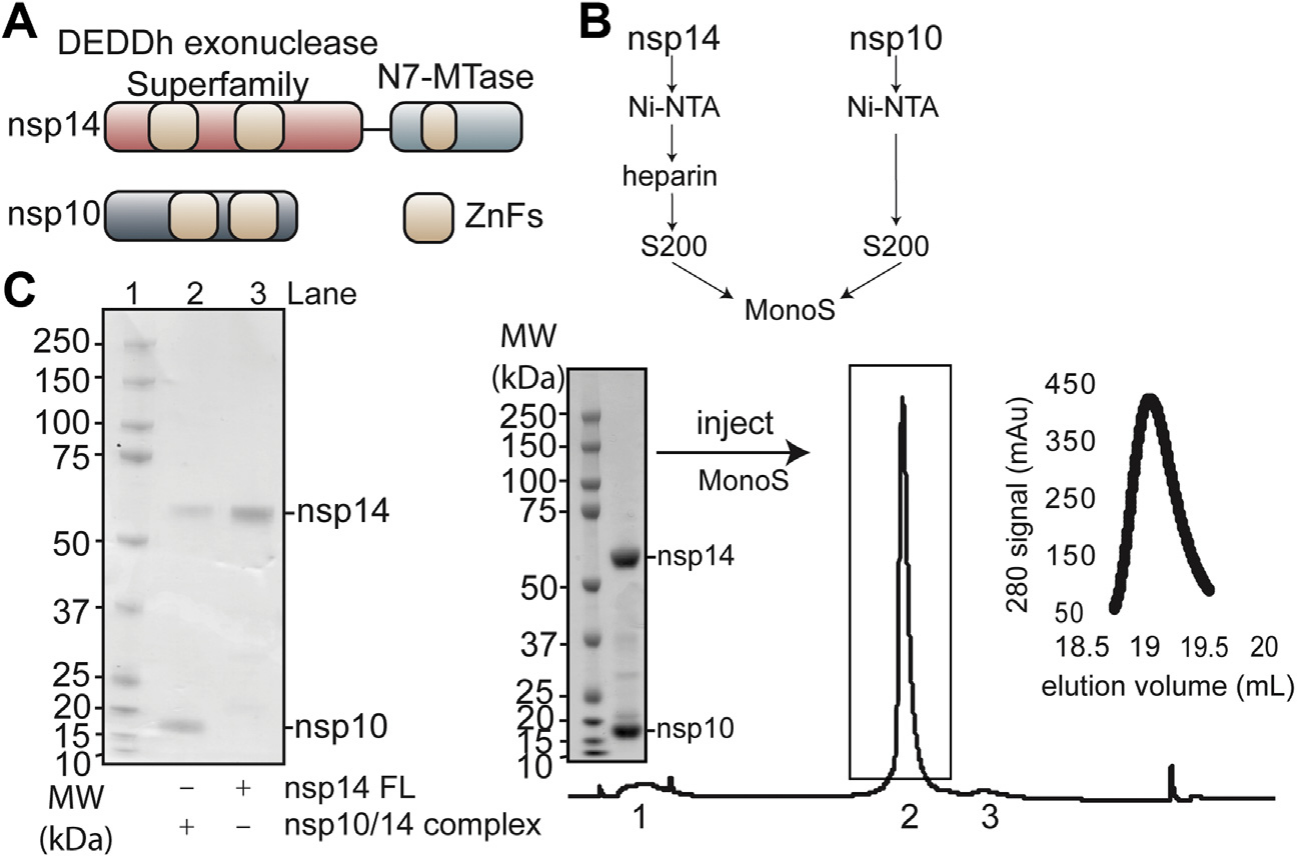
**NSP10 and NSP14 purifications for molecular complex formation.***A,* schematic of NSP14 and NSP10 domain structures. *B,* His-tag NSP10 and His-tag NSP14 chromatography purification schemes. Schematic of individually and combined to load on Mono S 5/50 GL to form NSP10/NSP14 complex. *C, left,* SDS-PAGE gel of His-NSP14 full length (FL) and His-NSP10/14 complex purifications. Lane 1, marker; lane 2, stoichiometric complex NSP14 (*upper band*) and NSP10 (*lower band*); and lane 3, NSP14 FL. *Middle,* 4 to 12% SDS-PAGE Coomassie-stained gel of NSP10 and NSP14 loaded onto Mono S 5/50 GL column. *Right,* chromatograph of Mono S 5/50 GL coelution of NSP10 and NSP14, peaks labeled 1 to 3. Peak 1 is excess NSP10, peak 2 is NSP10/14 complex, and peak 3 is excess NSP14 (Fig. S2*D* for corresponding SDS-PAGE). Inset is a zoom of peak 2 from the Mono S 5/50 GL defining coelution of NSP10/14 complex. DEDD, residues forming the active site aspartic acid (D) and glutamic acid (E); hDEDD, h indicates human; N7-MTase, guanine-N7-methyltransferase; NSP, nonstructural protein; ZnF, zinc finger domain.

At the writing of this publication, numerous publications have highlighted the difficulties encountered expressing full-length (FL) NSP14 in quantities required for structural studies in the absence of fusion peptides or coexpression with other NSP proteins. As noted by other studies, we were also unable to coexpress NSP10 and NSP14 in stoichiometric or near stoichiometric amounts (Fig. S1) (11, 16, 17). In addition, tethering two proteins on a single polypeptide can generate artificial or nonbiologically relevant complex interactions or altered domain interfaces. Structural and enzymatic studies of NSP10/14 and other protein complexes can be significantly altered by large expression tags and nonstoichiometric coexpression of complexes, as demonstrated in Ref. (17). Overall, the lack of straightforward purification protocols of NSP10 and NSP14 has hampered biochemical and structural studies of this important SARS-CoV-2 complex. To this end, the mechanism and specificity of regulation in SARS-CoV-2 of NSP10 enhances exonuclease activity of NSP14, and the incorporation of metabolic reaction byproducts such as S-adenosyl-l-methionine/SAH or remdesivir monophosphate (RMP) is unknown.

Here, we present a straightforward bacterial expression and *in vitro* purification scheme to produce NSP14 and NSP10 individually in quantities useful for structural studies. While advantageous for future studies, individually purified NSP14 and NSP10 enabled the kinetic characterization to define the enzymatic advantage of NSP10 on NSP14 exoribonuclease activity. We observed that NSP10 increases both the catalytic efficiency of NSP14 exoribonucleolytic activity by up to 260-fold and enhances the overall protein thermal stability. Furthermore, we determined that, unlike other exonucleases, NSP14 exonuclease activity is not inhibited by the monophosphate (MP) intermediate of remdesivir. These purification methods and biochemical analyses begin to lay the groundwork for future structural studies defining the mechanism of SARS-CoV-2 viral synthesis as well as designs for future therapeutic treatments.

## Results

### NSP14 and NSP10 individually and *in vitro* copurified complex aid structural and biochemical studies

The difficulty around the expression and purification of NSP14 FL has led to a lack of thorough biochemical analyses. Here, we report a new method for the expression and purification of both NSP10 and NSP14, individually, in quantities sufficient for structural studies in the absence of fusions, coexpressions, or variable tags. This approach has allowed us to probe the effect of NSP10 on NSP14 FL. Briefly, we grew bacterial Rosetta2 pLacI (DE3) competent cells with plasmids for either pDest527 (N-terminal His-tag) NSP10 or NSP14 FL in terrific broth (pH 7.2) media, 3 and 6 l, respectively. Cultures were inoculated and grown at 37 °C to an absorbance of 2 at 600 nm, accomplished in approximately 4 h, and induced with the addition of 0.2 mM IPTG. NSP10 bacterial cultures were grown for another 4 h at 37 °C and harvested at 4000 rpm for 25 min. In comparison, overnight expression of NSP10 led to a substantial decrease in final protein yield (data not shown). NSP14 cultures were grown overnight at 16 °C.

Purification of NSP10 and NSP14, individually, was carried out *via* a relatively straightforward two (NSP10) or three (NSP14)-column purification scheme: (cytiva columns) (i) 5 ml nickel (Ni)–nitrilotriacetic acid (NTA) HP, (ii) 5 ml heparin HP (NSP14 only), and (iii) S200 increase. To achieve the most highly purified proteins, Ni–NTA HP was loaded with Ni buffer (25 mM HEPES [pH 8], 0.5 mM Tris(2-carboxyethyl)phosphine [TCEP], 500 mM NaCl, and 0.5% Nonidet-P40 [NP-40] supplemented with 40 mM imidazole) and washed in excess of 10 column volumes (CVs) with Ni buffer and then followed by Ni buffer with no NP-40 to remove NP-40 from future steps of purification. The protein of interest was eluted in a stepwise elution with 2 to 5 CVs at each step (steps: 62.5, 135, and 250 mM imidazole). Fractions containing NSP10 and NSP14 were identified through SDS-PAGE and Coomassie staining and pooled (Fig. S2). After optimization, it was determined that NSP14 benefited from a three-column purification. Interaction to heparin can be improved by decreasing ionic strength; consistent with this, NSP14 binding to a heparin requires a reduction in NaCl (50 mM used for this study) for binding sufficiently with negligible protein loss. Dilution of the protein 10-fold to achieve 50 mM NaCl with 25 mM HEPES (pH 8) and 0.5 mM TCEP (dilution buffer) did not result in visible changes to solution solubility (Fig. S2). Protein was eluted over a linear gradient elution from 50 mM NaCl, 25 mM HEPES (pH 8), 0.5 mM TCEP (heparin buffer A) to 1 M NaCl, 25 mM HEPES (pH 8), and 0.5 mM TCEP. The single peak of NSP14 generally eluted between 180 and 250 mM NaCl but were confirmed by SDS-PAGE and Coomassie staining. Heparin fractions containing NSP14 were concentrated and buffer exchanged during concentration with 250 mM NaCl, 25 mM HEPES (pH 8), and 0.5 mM TCEP (S200 buffer). Buffer exchanging into the S200 buffer reduces nonspecific binding to the S200 increase column. The individual proteins were then concentrated to 500 μl to 2 ml and loaded on an S200 increase for size-exclusion chromatography (SEC) in S200 buffer. Both NSP10 and NSP14 contained additional protein contaminants prior to SEC; after S200, only fractions containing the purest protein of interest were pooled and buffer exchanged into a 150 mM NaCl, 25 mM HEPES (pH 8), and 0.5 mM TCEP buffer. For a more thorough step-by-step purification protocol, refer to the Experimental procedures section and Figure S2. This purification method yielded near homogeneous protein as observed on a Coomassie-stained SDS-PAGE gel (Figs. 1*C* and S2). Also, noteworthy, individually, NSP14 and NSP10 and the complex were concentrated in excess of 10 mg l^−1^ without visible effects to solubility.

After purifying the individual proteins, as described previously (Fig. 1*B*), NSP10 and NSP14 were combined, with NSP10 in 10-fold molar excess (Fig. 1*C*). This protein mixture was then diluted to 50 mM NaCl final concentration using dilution buffer and loaded on a Cytiva Mono S 5/50 GL column. NSP14 and NSP10 can be concentrated before loading onto the Mono S at 50 mM NaCl without compromising solubility or final protein concentration. The mono S column was run at 10 ml/min, washed with buffer A (5 CVs), and eluted with a linear gradient over 15 CVs from 50 mM NaCl to 1 M NaCl. The NSP10 isoelectric point (pI = 7.95) is such that it does not bind tightly to the Mono S resin in the buffering conditions used. Therefore, the majority of the 10-fold excess NSP10 will either flow through the column during loading or will elute very early in the column wash or linear gradient wash (Fig. 1*C*, peak 1). The second peak of the elution represents the formed complex, consisting of near stoichiometric amounts of NSP14 and NSP10 (Fig. 1*C*, peak 2). The complex charge is sufficiently different from either individual protein such that the complex elutes before free NSP14 (Fig. 1*B*, peaks 1 and 3). Given that NSP10 was in 10-fold excess, very little unbound NSP14 eluted. The stoichiometric pure complex achieved by this technique is unlike that formed by prior methods for the SARS-CoV-2 NSP10/14 complex (11, 18, 19). Final yields of the complex from the Mono S column are approximately 0.6 mg l^−1^ of culture. We believe this method of expression and purification will further enhance the capabilities to perform ongoing studies of replication/transcription complex and molecular mechanisms underlying capping the nascent SARS-CoV-2 mRNAs.

### NSP10 imparts protein stability and exonuclease activity of NSP14

**Figure 2:**
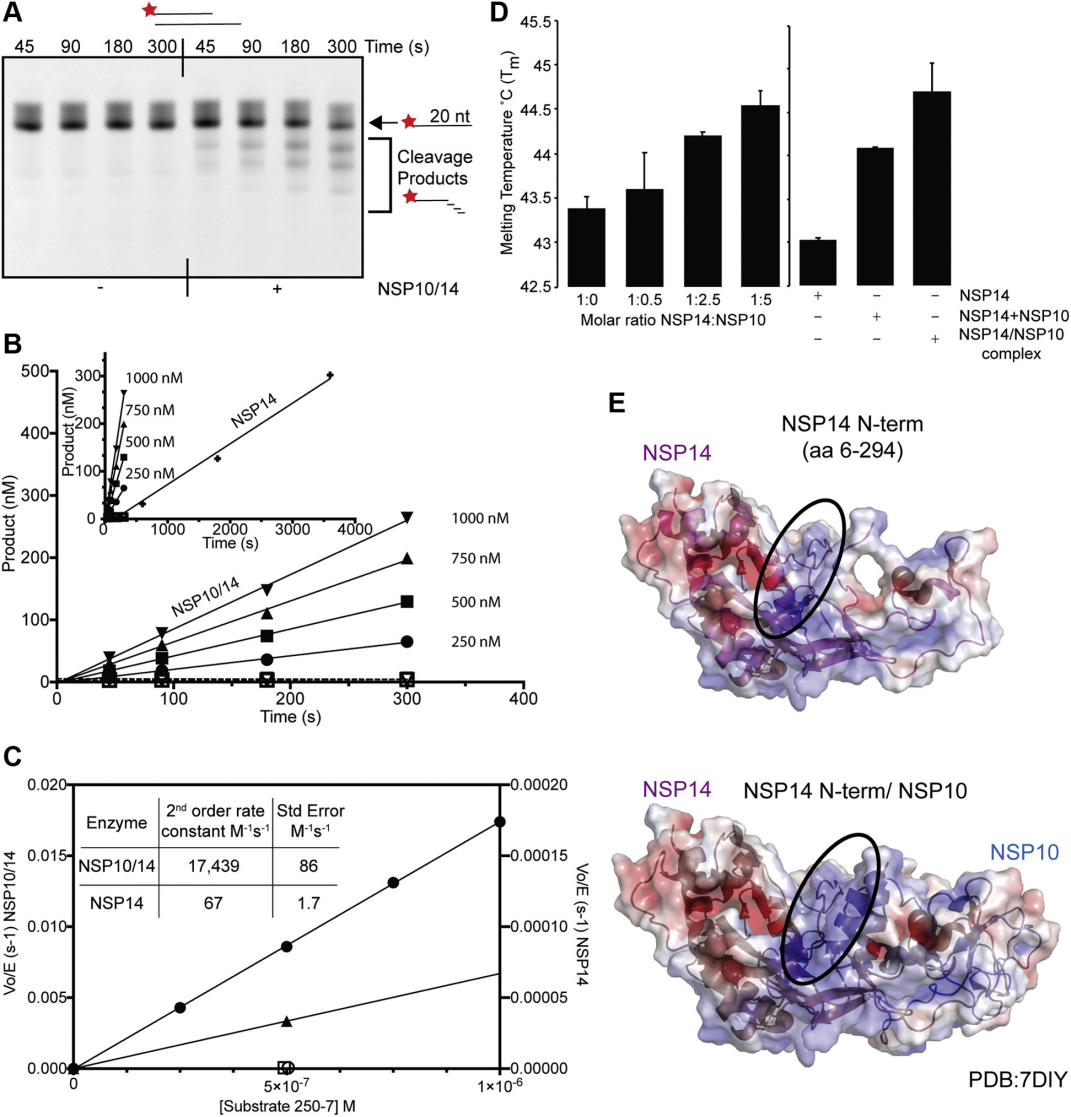
**NSP10 enhances NSP14 stability and exonuclease activity.***A,* sample Urea-PAGE gel of NSP10/14 exoribonuclease activity. The substrate, a 20 base pair duplex with an 8 bp overhang fluorophore-labeled RNA primer at 500 nM, is shown along the top of the gel. About 5 min time course of control samples (no enzyme) lanes 1 to 4 and NSP10/14 (50 nM) lanes 5 to 8. *B,* main graph: Exonuclease progress curves for the NSP10/14 complex (50 nM) and increasing RNA substrate, as indicated in the figure. Standard error, if larger than symbols, is shown with error bars. *Open squares* and *open triangles*, both near the *X*-axis, represent 500 and 1000 nM substrate controls in the absence of NSP10/14. Inset graph: because of slower turnover, NSP14 (2.5 μM) activity was measured at the time points indicated. *C,* plot of initial rates, determined from the progress curves in *B*, with respect to substrate concentration: *Closed circles* represent NSP10/14 initial rates (*left*, *Y*-axis). Isolated NSP14 initial rates (*right*, *Y*-axis) are illustrated with *open circles* for 50 nM enzyme, *open squares* for 500 nM enzyme, and *closed triangles* for 2.5 μM enzyme. The three NSP14 initial rate points correspond to 500 nM substrate; however, *open circles* and *open squares* have been slightly offset along the *X*-axis for clarity. The table inset displays the second-order rate constants of the NSP10/14 complex and isolated NSP14 (1.74 × 10^4^ ± 8.6 × 10^1^ and 6.70 × 10^1^ ± 1.7 × 10^0^ M^−1^s^−1^, respectively). The second-order rate constant for NSP10/14 was calculated using linear fits of the initial rates observed across varying substrate. The second-order rate constant for isolated NSP14 was calculated both through a linear fit of the initial rates measured at three different enzyme concentrations with constant substrate as well as the fit of the initial rate measured with 2.5 μM enzyme from the origin to the 500 μM substrate point, and the results were found to be within the stated error range of one another. *D,* differential scanning fluorimetry (DSF): *left,* titration of NSP10 (0, 1, 5, and 10 μM) in the presence of NSP14 (2 μM). *Right,* NSP14 (DMSO-containing) stabilization complex by *in vitro* mixing or copurification with NSP10. All error bars are standard deviation. *E,* the electrostatic potential of NSP14 and NSP10/14 (Protein Data Bank ID: 7DIY) was calculated with Pymol plug-in APBS with electrostatic isocontours of ±10 kT (*blue* and *red*, respectively). A *black oval* identifies an acidic patch enhanced upon association of NSP10/14. DMSO, dimethyl sulfoxide; NSP, nonstructural protein.

With our purified NSP10 and NSP14 and the stoichiometric complex, we were uniquely positioned to perform biochemical characterization of both components individually as well as assembled and purified as an NSP10/14 complex. In SARS-CoV-1, it was shown that NSP10 enhances the exonuclease activity of NSP14 (20, 21). However, it was unknown to what degree NSP10 enhanced the exonuclease activity of the SARS-CoV-2 NSP14 FL. To address this question, the 3′ exonucleolytic activity of NSP14 was measured alone and within the NSP10/14 complex. Exonuclease activity was measured on a 5′ Cy5-labeled 20-mer RNA primer annealed to an unlabeled 28-mer RNA complement. An 8-nucleotide single-stranded overhang exists at the 5′ of the complement strand (Fig. 2*A*). The NSP10/14 complex (50 nM) catalyzed digestion of this labeled RNA primer over a 5-min time course (Fig. 2*A*). While the substrate alone showed no quantifiable degradation in the absence of NSP10/14 (Fig. 2*A*, lanes 1–4), the presence of the nuclease complex leads to substantial cleavage of the substrate. The cleavage products, which follow a laddering pattern, are most clearly visible at both 180 and 300 s time points (Fig. 2*A*, lanes 7–8). NSP10/14 exonuclease activity was then measured over a range of substrate concentrations (250 nM–1 μM) (Fig. 2*B*). The activity of NSP14 (50 nM) alone was undetectable over this time course. Therefore, NSP14 activity was also measured at 500 nM and 2.5 μM protein concentrations. Furthermore, the activity of NSP14 alone was measured at time points longer than was required for NSP10/14 complex (Fig. 2*B*, inset). Because of a slower rate of cleavage product formation, a 60 min time course was used to yield comparable amounts of product. Initial rates (*V*_o_) were determined from these reaction progress curves and plotted in Figure 2*C* with respect to substrate concentration. Initial rates for the NSP10/14 complex and NSP14 alone are plotted on the *Y*-axis (left and right, respectively). In Figure 2*C*, the plot demonstrates that initial rates are linearly dependent on substrate concentration. For NSP10/14, we determined a *k*_cat_/*K*_*m*_ value of 1.74 × 10^4^ ± 8.6 × 10^1^ M^−1^s^−1^, and for NSP14, we determined a *v/K* rate constant of 6.70 × 10^1^ ± 1.7 × 10^0^ M^−1^s^−1^. Thus, the nuclease activity of NSP14 is strongly activated by NSP10, with a 260-fold increase in the second-order rate constant for the NSP10/14 complex compared with NSP14 alone. Attempts were made to reconstitute the activity of NSP14/NSP10-purified complex using individually purified NSP14 + NSP10. However, this approach was unsuccessful, consistent with observations made by Canal *et al.* (17).

To further probe the mechanism of the NSP10-dependent stimulation of NSP14 exonucleolytic activity, we employed differential scanning fluorimetry (DSF) to assess changes to the thermal stability of NSP14. We first determined the melting temperature (*T*_m_) of NSP14 alone to be 43.4 ± 0.3 °C. A titration of NSP10 over the molar ratios 1:0, 1:0.5, 1:2.5, and 1:5 demonstrated an increase in thermal stability, with a maximum *T*_m_ of 44.5 ± 0.2 °C occurring at a 1:5 ratio (Fig. 2*D*). Through additional experimentation, in the presence of DSF dimethyl sulfoxide (DMSO) buffer, we also compared thermal stabilities of a 1:10 molar ratio of the NSP10 + 14 reconstituted complex and a copurified complex at near stoichiometric ratios. The *T*_m_ of these two complexes is 44.1 ± 0.01 °C and 44.7 ± 0.3 °C, respectively, whereas NSP14 alone is 43.0 ± 0.01 °C (Fig. 2*D*, right). NSP10 also reproducibly stabilized NSP14 in buffer conditions that do not contain DMSO (Fig. S3). While preparing this publication, an X-ray crystallography structure of NSP10 and NSP14 N terminus DEDD domain (residues 6–294) was published (Protein Data Bank ID: 7DIY) (19). The solvent-exposed surface of NSP14, specifically the region in close proximity to the NSP10 binding site, is composed primarily of positively charged residues (Fig. 2*E*, left). When NSP10 and NSP14 form a molecular complex, as displayed in the crystal structure (Fig. 2*E*, right), the solvent-exposed positively charged area increases. The change in electrostatic potential of the complex likely accounts for an earlier elution of the complex from a Mono S column under a linear NaCl gradient. Taken together, the enhanced positive solvent-exposed interface and the increased thermal stability imparted by NSP10 within NSP10/14 complex support the strong activation of NSP14 exonuclease activity in the presence of NSP10.

### NSP10/14 is insensitive to remdesivir MP but stabilized by SAH

**Figure 3:**
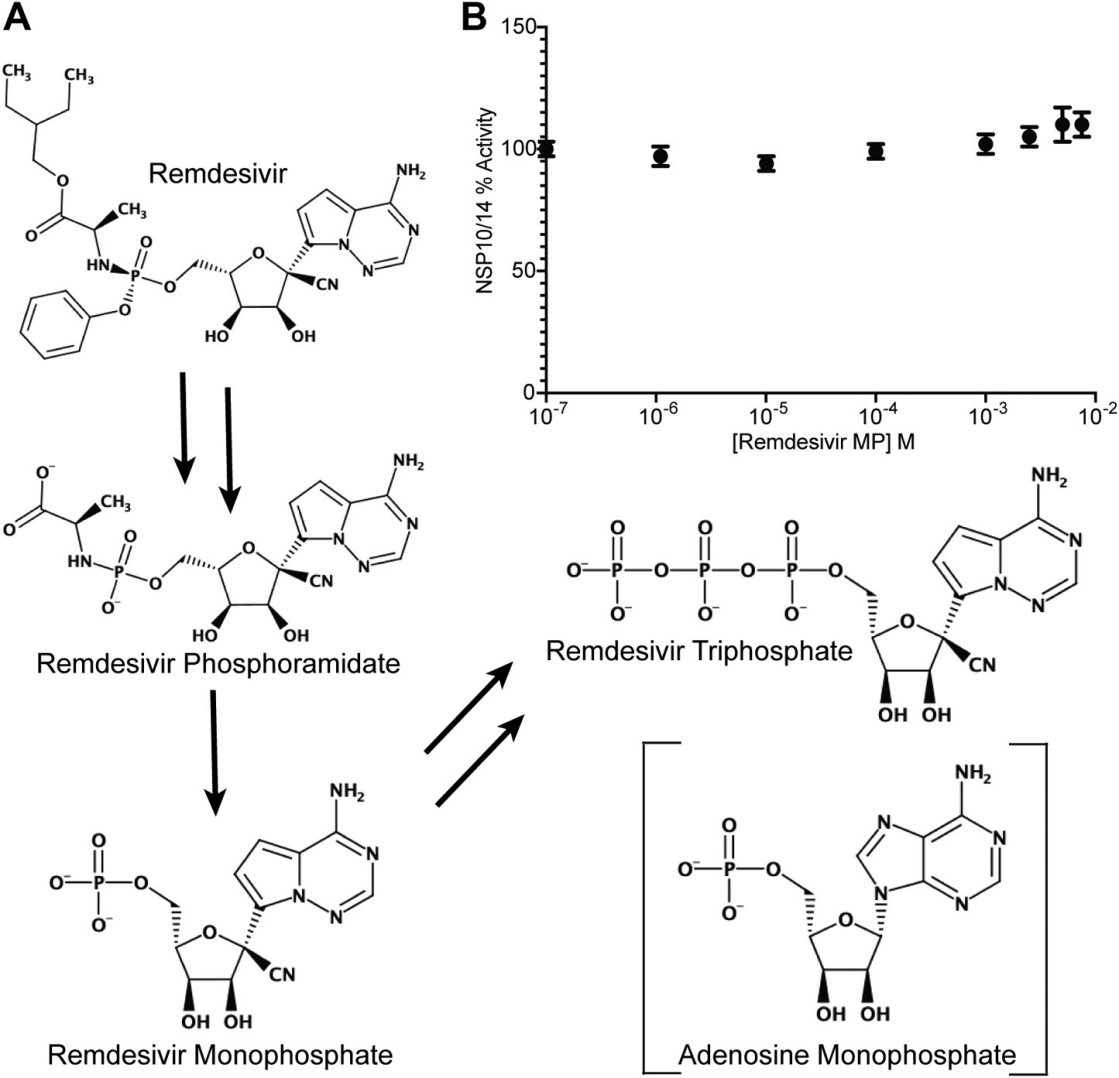
**Remdesivir monophosphate (RMP) does not inhibit NSP10/14.***A,* schematic of the metabolic activation of remdesivir. RMP is an intermediate in this pathway and is a close structural analog to AMP, which is shown in brackets. *B,* NSP10/14 (50 nM) exonuclease activity on 250 nM substrate in the presence of 0 to 7.5 mM RMP (standard error of initial rate fits over six time points shown with *black bars*). NSP, nonstructural protein.

Remdesivir is a prodrug that must be metabolized *in vivo* into remdesivir triphosphate to yield the active drug (Fig. 3*A*). Biochemical studies have shown that RNA-dependent RNA polymerase of SARS-CoV-2 can incorporate remdesivir triphosphate into the growing RNA chain. An intermediate in the activation of remdesivir is the conversion of a phosphoramidate to the MP form of the drug (22). RMP is a close mimic of AMP (Fig. 3*A*, AMP in brackets). Many antiviral nucleotide analogs, such as AZT, accumulate *in vivo* at the MP stage in very high concentrations (23, 24), and these nucleoside MPs can inhibit the intrinsic exonuclease activity of human polymerases (25). To investigate whether the exonuclease NSP10/14 is a secondary target of remdesivir, we measured nuclease activity in the presence of increasing RMP (Fig. 3*B*). No significant change in exonuclease activity was observed up to 7.5 mM RMP, suggesting that the MP form of this nucleoside analog, even in high concentrations, does not inhibit nuclease activity of NSP10/14.

**Figure 4:**
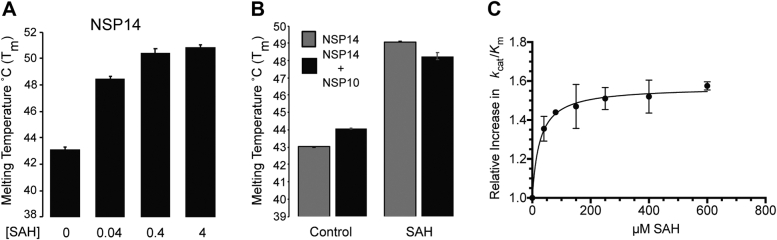
**NSP14 C-terminal engagement of S-adenosylhomocysteine (SAH).***A,* increase of NSP14 (2 μM) *T*_m_ in the presence of the product inhibitor SAH (0, 0.04, 0.4, and 4 mM) measured by DSF, 0 μM is DMSO control. *B,* SAH (0.4 mM) stabilization as compared with DMSO (control) condition of NSP14 (2 μM) (*gray bars*) compared with NSP14 (2 μM) 1:10 M ratio of NSP10 (*black bars*). *C,* SAH enhances the catalytic efficiency (*k*_cat_/*K*_*m*_) of the exonucleolytic activity of NSP10/14 (50 nM) with *K*_1/2_ = 25 ± 9 μM (standard error of initial rate fits over six time points, performed in duplicate, shown with *black bars*). DMSO, dimethyl sulfoxide; DSF, differential scanning fluorimetry; NSP, nonstructural protein.

In addition to the N-terminal exonuclease activity of NSP14, the C-terminal domain possesses MTase activity important for RNA capping (11). It is well documented that the MTase reaction product SAH is a nonspecific inhibitor of MTases (26). Consistent with this, it has been shown that SAH inhibits the MTase activity of NSP14 (27), yet it is unclear the impact SAH may have on the nuclease activity of NSP14. To address this issue, we first used DSF to determine the impact that SAH imparts on the thermal stability of NSP14 and NSP10/14 complex. We titrated SAH at 0, 40, 400 μM, and 4 mM (Fig. 4*A*). Interestingly, we observed a very substantial increase in NSP14 protein stability in the presence of SAH. In control conditions, the *T*_m_ of NSP14 is 43.1 ± 0.2 °C, and even at the lowest concentration of SAH (40 μM), the *T*_m_ of NSP14 is substantially increased to 48.5 ± 0.17 °C. Selecting the intermediate concentration (400 μM), we then added NSP10 in a 1:5 molar ratio to NSP14/SAH. This addition slightly reduces the thermal stability of NSP14/SAH from 49.1 ± 0.03 to 48.2 ± 0.2 °C (Fig. 4*B*). We also measured the exonuclease activity at increasing concentrations of SAH (Fig. 4*C*). SAH leads to an increase in *k*_cat_/*K*_*m*_, suggesting that SAH imparts a modest activation of NSP10/14 exonuclease activity, with a *K*_1/2,act_ = 25 ± 9 μM. This activation constant is consistent with the observed increasing *T*_m_ upon SAH binding from 0 to 40 μM and plateauing in the mid to high micromolar range.

## Discussion

Overall, the COVID-19 pandemic, the rapid spread of the SARS-CoV-2, and the deadly consequences of contracting SARS-CoV-2 have highlighted the need for additional and more specific viral inhibitors targeting coronaviruses. The primary target for nucleoside analog viral inhibitors is thought to be NSP12 and, to a lesser extent, NSP14 (28). It is also speculated that regulation of coronavirus genome fidelity may rely exclusively on NSP10/12/14 (29). Targeting NSP14 and NSP14 in complex with its coactivator NSP10, therefore, could prove to be a viable target in the treatment of COVID-19. Here, we have established a straightforward purification scheme, and, importantly, we have introduced a copurification strategy that can be used to generate an active stoichiometric protein complex for the SARS-CoV-2 exoribonuclease. Unlike the observed reconstituted exonuclease activity of SARS-CoV-1 NSP10/14 (30), we were not able to reconstitute NSP14 exonuclease activity by titrating NSP10. While we cannot rule out expression systems or protein affinity tag, others have noted that these proteins expressed and purified form *E. coli* were not active or able to stimulate (17) as compared with eukaryotic expression systems. We believe this highlights the utility of the stoichiometrically purified complex for SARS-Cov-2. To this end, *in vitro* purification has the potential to promote additional biochemical and structural analyses of the NSP10/14 complex and ultimately lead to a structure-guided approach aiding in the design of inhibitors.

A recent publication has shown a quaternary complex of NSP10, NSP14, NSP8, and RNA (18). In this complex, RNA is bound in the positive electrostatic patch that is formed upon NSP14/NSP10 complex formation (Fig. 2*E*, right). The positioning of nucleic acid is consistent with our analyses of NSP10 coactivator stabilizing the protein structure of NSP14 FL and increasing the N-terminal exonucleolytic activity. It is likely that the enhanced electrostatic potential provides a better compliment for the negatively charged RNA; however, to date, a thorough thermodynamics study of SARS-CoV-2 NSP14/NSP10 RNA binding has not been biochemically investigated. With the most recent structures and a mechanism for copurification, it would be interesting to generate mutations in the RNA-binding surface and the NSP10/14 interface to determine the molecular components that define this ternary complex. Given the increased *T*_m_, changes to the electronegative patch, and the increased second-order rate constant, it is tempting to speculate that the interface of NSP10/14 allows for enhanced RNA binding. We cannot eliminate the possibility that the NSP10 interface promotes an allosteric switch to the active site of NSP14, as was proposed for NSP10/NSP16 2′-O-MTase active site in Middle East Respiratory Syndrome-CoV and SARS-CoV-1 (13, 31).

NSP10 functions as an important cofactor to both NSP14 and NSP16, the 2′-O-MTase enzyme. NSP16 promotes the cap-1 structure adding a methyl group onto the cap-0 structure and is completely inactive in the absence of NSP10 in SARS-CoV-1 (30). It is proposed that NSP10 allosterically activates NSP16 and helps to assemble the MTase active site (32). Here, we observe that while NSP14 is capable of exoribonuclease activity, the activity is orders of magnitude lower than the activity of the NSP10/14 complex. In addition, NSP14 has a lower *T*_m_ in the absence of NSP10. The inherent instability may partially explain difficulties in obtaining an NSP14 Apo structure and the difficulties of *in vitro* purification for NSP14 observed in previous publications. Despite this, the binding interface of NSP10/14 and NSP10/16 significantly overlap, and future studies will be needed to determine the dynamic balance of NSP10 as an activator of both NSP14 and NSP16.

As the only Food and Drug Administration–approved antiviral therapeutic for COVID-19, it is critically important to understand the impact of remdesivir on multiple components of the SARS-CoV-2 core replication complex. While it is known that incorporation of remdesivir into a growing RNA by NSP12 causes delayed chain termination (15), it is unknown whether NSP14 is a secondary target of remdesivir. Remdesivir is supplied as a prodrug and must be metabolized *in vivo* to the active triphosphate drug. During this process, RMP is formed (Fig. 3*A*). Previous studies have demonstrated that chain-terminating antivirals may accumulate as nucleotide analog MPs, and that these compounds can directly inhibit the exonucleolytic proofreading function of host polymerases (25). In SARS-CoV-2, NSP14 functions as the replication proofreading exonuclease. We have shown here that RMP, even at low millimolar concentrations, does not inhibit NSP14 exonuclease activity. Given the current remdesivir dosage guidance (33), it does not appear that accumulated RMP inhibits NSP14 as a secondary target during treatment and may leave open the possibility of a dual-drug approach that also impedes NSP14 proofreading activity.

NSP14 is a dual function enzyme, with these separate functions being housed at either the N-(exonuclease) or C-(MTase) terminal domain. However, these two domains share an extensive interface (19). It has been shown that NSP14 C-terminal MTase activity does not require NSP10 and is, in fact, robust in the absence of NSP10 consistent with our observation that NSP14 is stabilized by SAH above the IC_50_ for inhibition of the C-terminal activity (16, 27). The C-terminal MTase activity of NSP14 has been targeted for small-molecule inhibitor design (17, 27). Surprisingly, we observed a small but significant increase in the exonucleolytic *k*_cat_/*K*_*m*_. Given this interplay, it would be advantageous to also perform exonuclease activity assays in the context of the FL protein when MTase inhibitors of NSP14 are considered.

The assembly of an *in vitro* and stoichiometric NSP10/14 complex presented here provides a foundation for future biochemical and structural investigations. We have used this to demonstrate that NSP10 strongly activates NSP14, both through exonuclease assays and thermal stability. We have also demonstrated that SAH, an inhibitor of the C-terminal MTase activity, further stabilizes NSP10/14 and activates N-terminal NSP14 exoribonuclease activity, potentially opening the door to crosstalk between the two distal NSP14 active sites. Finally, we have shown that NSP10/14 activity is not inhibited by RMP. In total, this investigation could lead to the design of additional antiviral therapeutics that may be used singly or in combination with other therapeutics, such as remdesivir, in the treatment of COVID-19 and potentially other coronaviruses.

## Experimental procedures

### Plasmids

pDONR223 SARS-CoV-2 NSP10 (Addgene; plasmid no. 141264; http://n2t.net/addgene:141264; Research Resource Identifier: Addgene_141264) and pDONR223 SARS-CoV-2 NSP14 (Addgene; plasmid no. 141267; http://n2t.net/addgene:1412647; Research Resource Identifier: Addgene_141267); both were a kind gift from Fritz Roth (34). Using Gateway LR technology (Invitrogen), NSP10 and NSP14 were subcloned individually into gateway competent expression vector pDEST527 (N-terminal His-tags NSP10 and NSP14) for bacterial expression.

### Protein expression and purification

#### Individual NSP10 and NSP14 purifications

pDEST 527 NSP14 and NSP10 were individually transformed into Rosetta2 pLacI (DE3) competent cells (Millipore) and grown to absorbance of ∼2 at 600 nm in terrific broth at 37 °C. *E. coli* cells were induced with 0.2 mM IPTG. NSP14 was induced at 16 °C overnight and NSP10 for 4 h at 37 °C. Pellets of both NSP10 and NSP14 were harvested (4000 rpm, 25 min). Pellets were stored at −20 °C. Thawed pellets were resuspended in Ni-loading buffer (25 mM HEPES [pH 8], 500 mM NaCl, 40 mM imidazole, 0.5 mM TCEP, 5% glycerol, 0.5% NP-40 supplemented with 1 mM PMSF, Roche EDTA-free protease inhibitor cocktail, and 1 mg/ml lysozyme) resuspension buffer volume used is 25 ml per 1 l of original culture. Resuspended lysates were stirred constantly at 4 °C for 30 min and then sonicated at 85% power on ice for approximately 3 min total run time with repeating rounds of 30 s sonication 30 s pause. Once lysates were homogenous and no longer opaque, lysates were cleared by centrifugation at 18,000 rpm for 60 to 90 min at 4 °C (ss-34 rotor). Cleared lysates on ice were loaded by peristaltic pump 4 to 5 ml/min onto Ni–NTA HP column. Protein was washed with Ni wash buffer in excess of 10 CVs (Ni loading buffer with no NP-40), and then eluted stepwise, used 62.5, 135, and 250 mM with elution buffer (Ni wash buffer supplemented with variable imidazole up to 250 mM imidazole). Eluted fractions containing proteins of interest as detected through SDS-PAGE and Coomassie staining were collected and pooled. NSP14 fractions were diluted to 50 mM NaCl and loaded onto a heparin column HP 5 ml. Proteins were eluted over a linear gradient with 15 CVs of gradient column (5 ml/min) buffer A (25 mM HEPES [pH 8], 50 mM NaCl, and 0.5 mM TCEP) and buffer B (buffer A supplemented to 1 M NaCl) 2 ml of elution per fraction. NSP10 was purified identically to NSP14 over Ni–NTA HP column, and fractions containing NSP10 were concentrated to 500 μl and a single injection loaded onto an S200 increase 10/300 GL for SEC (25 mM HEPES [pH 8], 250 mM NaCl, and 0.5 mM TCEP). SEC was also performed on NSP14, application dependent. Proteins were concentrated, and protein was flash frozen in liquid nitrogen and stored at −80 °C.

#### Purification of NSP10/14 complex

NSP10 and NSP14 were individually purified as described previously. Proteins were then combined with NSP10 in 10 M excess and diluted to 50 mM NaCl using heparin buffer A. The diluted complex was then loaded onto a Cytiva Mono S 5/50 GL. A gradient elution (15 CVs) was performed using buffer A and buffer B. Final buffer exchanged was during concentration to a storage buffer (25 mM HEPES [pH 8], 150 mM NaCl, and 0.5 mM TCEP). All proteins were flash frozen in liquid nitrogen and stored at −80 °C.

### Nuclease assay

5′ Cy5-labeled RNA primer 250 (5′/Cy5/AUAAUAGAUGAUCAAACGCG) (synthesized by IDT) was mixed at a 1:1.2 stoichiometric ratio with unlabeled RNA complement 257 (5′GCUUCUUCCGCGUUUGAUCAUCUAUUAU) (synthesized by Dharmacon) in 5 mM Tris–HCl (pH 7.5), 100 mM NaCl, and 50 μM EDTA. The oligonucleotides were heated to 90 °C and gradually cooled to 25 °C to facilitate annealing. Nuclease activity was assayed in 25 mM HEPES (pH 8.0), 25 mM NaCl, 1 μM bovine serum albumin, 1 mM TCEP, 0.1 mM EDTA, and 10 mM MgCl_2_. The NSP10/14 complex was measured at 50 nM enzyme complex and NSP14 at 50 nM, 500 nM, and 2.5 μM enzyme. Reactions were assembled on ice and transferred to 37 °C for 1 min before being initiated by addition of RNA oligonucleotide. Activity was measured with RNA concentrations over a range from 250 nM to 1 μM. Reaction time points were stopped in a quench solution containing 95% formamide, 15 mM EDTA, and 0.01% bromophenol blue. Time points were heated to 90 °C for 1 min, cooled to 75 °C, and loaded into 16% polyacrylamide gels with 8 M urea to separate products from the substrate. Fluorophore-labeled RNA was measured with an Amersham Typhoon 9000. Bands were quantified with ImageQuant software (Cytiva), and the percentages of substrate and product were calculated for each lane.

#### NSP10/14 activity with remdesivir and SAH

Remdesivir nucleoside MP (MedChemExpress) was dissolved in ultrapure water at 100 mM and shielded from light. SAH (Cayman Chemical) was dissolved in 100% DMSO at 70 mg/ml. Nuclease assays were performed as aforementioned, with the following modifications. NSP10/14 was held constant at 50 nM, and substrate was held constant at 250 nM. Varying concentrations of either RMP or SAH were preincubated with enzyme in the reaction mixture for 5 min at room temperature before being transferred to 37 °C. As before, reactions were initiated by addition of the RNA oligonucleotide.

### Protein thermal shift assay

DSF experiments were performed using 2 μM NSP14 and NSP10 concentrations as indicated in the relevant figure. Fluorescence emission was measured as the temperature was increased from 25 to 95 °C on a QuantStudio RT–PCR using Quant-Studio Real Time PCR system software. Reactions were conducted in 25 mM HEPES (pH 8.0), 60 mM NaCl, 0.1 mM TCEP buffer. Figures 2*D* (*left*) and 4, *A* and *B* contain 2.4% DMSO. DMSO was added during experimentation that included SAH, as a condition matching. Data were interpreted, and *T*_m_ values were calculated using Applied Biosystems Protein Thermal Shift Software. Two or more independent experiments were performed. A Boltzmann sigmoid was fit to the data to determine *T*_m_ values shown, and error bars are standard deviation.

## Data availability

All data are contained within the article and in the supporting information.

## Supporting information

This article contains supporting information.

## Conflict of interest

The authors declare that they have no conflicts of interest with the contents of this article.
