# Supplementary material for: Activation of the SARS-CoV-2 NSP14 3′–5′ exoribonuclease by NSP10 and response to antiviral inhibitors
Source: J Biol Chem. 2021 Dec 20;298(1):101518. doi: 10.1016/j.jbc.2021.101518 (PMC8685350; doi:10.1016/j.jbc.2021.101518)
Supplement: Supporting information [file mmc1.docx]

**Supporting Information:**

**Activation of the SARS-CoV-2 NSP14 3'-5' exoribonuclease by NSP10 and response to antiviral inhibitors**

Running title: SARS-CoV-2 and NSP10/14 activation

Amanda A. Riccio^+^, Eric D. Sullivan^+^, and William C. Copeland*

Supplementary Figure 1: NSP10 and 14 coexpression is insufficient for stoichiometric complexes. NSP10 and NSP 14 were cloned into pSF1 vector for coexpression using LR cloning technology. Cloning places a C-terminal his tag NSP 14 and an untagged NSP 10. NSP 14 was cloned into the vector closer to the start site and with a tag since it has weaker overall expression. Comparison of soluble and elution from a Ni column in a step wise elution in expressed in BL21DE3 and Rosetta2 cells. E1-E6 indicate the fraction of elution, where each numerical elution was 1 CV elution from the Ni column. Elution buffer is 500 mM NaCl, 25 mM HEPES pH 8, 0.5 mM TCEP supplemented with 62.5 mM (E1-E2), 125 mM (E3-E4), 250 mM (E5-E6) imidazole. Cells were grown, lysed, cleared, and Ni column purification was performed as described for figure 1 in the main text and “Experimental Procedures”.

Supplementary Figure 2: SDS-PAGE and Coomassie staining of representative purifications of his-tag NSP10 and his-tag NSP14 individually and his-tag NSP10/14 reconstituted complex. Steps in the individual NSP10 and NSP14 purifications in A-D are labeled in chronological order of purification and black boxes around protein bands indicate pooled fractions used to continue to the next purification step. Ni-affinity purification (A), Heparin column (applicable to NSP14 only) (B), S200 increase (C), Mono S (complex only) (D). A. Ni-affinity purification (left) NSP 14 and (right) NSP 10. Stepwise elution in the presence of increasing imidazole. At each step of elution, all volumes are in excess of 2CV. B. Heparin HP 5mL linear gradient elution (15CV) from 50 mM to 1 M NaCl with 2 mL elution fractions volumes. C. Injection of 0.5mL on S200 increase in 25 mM HEPES, 250 mM NaCl, 0.5 mM TCEP isocratic elution of 1CV at 0.6 mL/min

D. copurification of NSP10 and NSP 14 on Mono S column 15CV 50 mM to 1M linear gradient elution. Abbreviations: M =marker, MW=molecular weight, ND=not determined. ++/** concentrated greater than 10 mg/mL or 5 mg/mL, respectively, and concentration was stopped because of experimental requirements rather than because of solubility.

Supplementary Figure 3: Thermal stability of NSP14 in the presence and absence of NSP 10. Panels above were essentially performed as described in the Experimental Procedures. A. (left) plot of melting temperature of duplicate plots of NSP14 and NSP14/10 complex. Conditions are 25 mM HEPES pH 8.0, 0.1 mM TCEP buffer, 50 mM NaCl. (Right). B. Plot of NSP14 and NSP10/14 conditions 25 mM HEPES pH 8.0, 0.1 mM TCEP buffer 50 mM vs 25 mM of NaCl. C. Melting Temperature plots of NSP10 titrations performed in duplicate. Signal increases as NSP10 concentration increases, possibility owing to the instability of NSP10 in the absence of a binding partner and/or a higher order NSP10 formation.
